# Independent Origin of Phenol Non-responsive Phenotype Caused By *Phr1* Variation During Domestication of Asian and African Rice

**DOI:** 10.1186/s12284-026-00884-x

**Published:** 2026-01-22

**Authors:** Thet Htar San, Kokoro Iguchi, Daichi Ujiie, Shuhei Okada, Intan Widia Santika, Kiwamu Hikichi, Yoshiyuki Yamagata, Daisuke Fujita, Maria Stefanie Dwiyanti, Yuji Kishima, Yohei Koide

**Affiliations:** 1 Research Faculty of Agriculture, Hokkaido University, Sapporo, Japan; 2 Faculty of Agriculture, Kyushu University, Fukuoka, Japan; 3 Faculty of Agriculture, Saga University, Saga, Japan

**Keywords:** African rice, Domestication, *Oryza glaberrima*, Phenol color reaction, *Phr1*, Polyphenol oxidase

## Abstract

Phenol color reaction has been used to distinguish between two subspecies of Asian rice (*Oryza sativa*), *indica* and *japonica*. The trait is controlled by one single *Phr1* gene, which encodes a PPO enzyme that catalyzes the oxidation of phenolic compounds into brown or black pigments upon contact to phenol solution. In *O. sativa*, ssp. *indica* responds to phenol chemical assay by altering the rice hull color to black, whereas ssp. *japonica* remains unaffected due to mutations that render the gene non-functional. Although the different characteristics between subspecies in Asian rice is well known, there is no information about the variation of this trait in African rice, *Oryza glaberrima*, which was originated and domesticated independently of Asian rice. In this study, we found both phenol negative and positive lines in *O. glaberrima* and its wild ancestor *O. barthii* and detected the responsible non-functional mutation (1-bp deletion) in the exon 1 of the *Phr1* gene. Geographical distribution of its haplotype suggested that this mutation originated in *O. barthii* in Mali and was later inherited by *O. glaberrima*. The predominance of the non-functional *Phr1* alleles in *O. glaberrima* lines and the occurrence of the identical haplotypes in negative group of both *O. barthii* and *O. glaberrima* suggest that the negative phenol reaction was favored during domestication and breeding selection. The presence of a selection event is also supported by low nucleotide diversity of *Phr1* locus. However, genetic diversity of *Phr1* persists in African rice germplasm, as the functional alleles are still present in *O. glaberrima*. We also compared the nucleotide diversity of *Phr1* in African rice with that in Asian rice and found that their origins of the phenol responsive phenotype are independent. These findings expand the current understanding of African rice domestication and offer the valuable molecular marker for improved rice breeding.

**Supplementary Information:**

The online version contains supplementary material available at 10.1186/s12284-026-00884-x.

## Background

Rice (*Oryza spp.*) is one of the most important staple crops globally, feeding more than half of the world’s population and predominantly consumed in Asia. Although not originally a major crop in all African regions, it has become increasingly important, especially in urban and peri-urban areas where dietary preferences are shifting toward rice-based staples (Hegde and Hegde. 2013). The origins of agriculture in Africa were closely associated with the cultivation of African rice (*Oryza glaberrima* Steud.) along with other cereal crops such as sorghum and pearl millet (Doebley et al. 2006). In contrast to Asian rice (*Oryza sativa* L.), which holds a prominent position in global production, African rice stands out for its unique origins and genetic variability (Chang. 1976). African rice harbors significant genetic capabilities in terms of its resistance to both biotic and abiotic stress factors, making it a valuable reservoir of genetic traits for enhancing rice cultivars (Wambugu et al. 2013).

Domestication is a major factor influencing genetic variation in cultivated crops, often leading to a reduction in genetic diversity as a result of population bottlenecks during the domestication process. The domestication of *O. glaberrima* occurred approximately 3,000 years ago and it was independent of *O. sativa*, which originated in Asia around 9,000–10,000 years ago (Portères. 1976; Vaughan et al. 2008). Although *O. glaberrima* was the predominant rice species cultivated in West Africa prior to the fifteenth century, *O*. *sativa* was introduced through multiple historical routes, including trans-Saharan trade, Arab traders along the East African coast, and Portuguese and other European colonists via the Atlantic (Vaughan et al. 2008; Coclanis. 2003). Therefore, *O. glaberrima* was gradually displaced by *O. sativa* in many regions due to the latter's higher yield potential and broader agronomic adaptability, despite the former's superior resilience to local conditions (Linares. 2002). The African wild rice species *Oryza barthii* is recognized as the progenitor of *O. glaberrima*, through genetic and archaeological evidence (Duan et al. 2007; Huang et al. 2015; Stein et al. 2018; Wambugu et al. 2015; Wang et al. 2014). Whole-genome analyses of *O. glaberrima* and *O. barthii* indicate that the primary center of domestication was located in the Inland Niger Delta, particularly in northern Mali (Cubry et al. 2018).

Both Asian and African rice species depict a similar feature of domestication traits, *e.g.* the reduction of seed shattering, reduction of awn length and erect plant architecture, despite their independent origins. In Asian rice, the major genes that controls seed shattering are *sh4* (Li et al. 2006), *qSH1*(Konishi et al. 2006), *OsCPL1* (Ji et al. 2010), *OsSh1* (Lin et al. 2012), and *SH5* (Yoon et al. 2014). In African cultivated rice (*O. glaberrima*), orthologs of these genes also play a role in seed shattering, but with different mutations site in *OgSh4/GL4/SH3* (Wang et al. 2014; Wu et al. 2017; Win et al. 2017) and *SH5* (Cubry et al. 2018). In addition, *ObSH3* (Lv et al. 2018) is absent in African cultivated rice, demonstrating that similar non-shattering phenotypes arose from distinct genetic mechanisms. In Asian rice, awn development is regulated by two genes, *Regulator of Awn Elongation 1* (*RAE1*), *RAE2 and RAE3*, with loss-of-function mutations resulting in an awnless phenotype (Furuta et al. 2015; Bessho-Uehara et al. 2016). In *O. glaberrima*, these genes are functional but do not affect the awn development; instead, awn formation in African rice is controlled by the *RAE3* gene, located on chromosome 6 (Furuta et al. 2015). This trait in rice independently arises in Asian and African lineages through selection on different genes during parallel domestication (Furuta et al. 2015).

Erect plant architecture was selected for higher yield and planting density. In Asian rice, this was mainly caused by loss of *PROG1* function (Tan et al. 2008), while in African rice, genomic deletions encompassing *PROG1* and its paralog *PROG7*, both encoding C2H2-type zinc finger transcription factors regulating plant architecture, led to loss of *PROG7* expression, the key regulator of tiller angle in this species (Wu et al. 2018; Hu et al. 2018). These findings indicate that Asian and African cultivated rice underwent independent yet convergent domestication processes, where similar phenotypes evolved through selection on either the same genes with independent mutations or distinct genetic loci.

In *O. sativa*, there are four key criteria distinguishing between ssp. *indica* and ssp. *japonica* rice varieties: the rice hull color response to phenol solution, resistance to KCLO_3_, cold tolerance, and the length of hair on glume tips (Oka. 1953, 1958; Oka and Chang. 1961; Morishima. 1992). These phenotypic differences have also been proposed to reflect selective pressures associated with the domestication process (Oka and Chang. 1961; Chang. 1976). For one of the key criteria, phenol color reaction, the ssp. *japonica* types exhibit no color shift (a negative response), whereas ssp. *indica* varieties and Asian wild species, *Oryza rufipogon*, develop a dark brown or black color, attributed to the activation of polyphenol oxidase (PPO) enzymes (a positive response) (Oka. 1953). For governing the phenol color reaction in rice, the responsible gene is the *Phr1* on chromosome 4, with variations in this gene leading to discernible differences in both the intensity and type of color change observed (Yu et al. 2008). The phenol negative response characteristic of ssp. *japonica* varieties has been attributed to three distinct loss-of-function mutations at the *Phr1* gene which encodes the PPO enzyme, a member of the tyrosinase enzyme family (Yu et al. 2008).

Phylogenetic and population genetic analyses suggest that loss-of-function mutations in *Phr1* were rapidly fixed in ssp. *japonica* through positive selection, likely driven by human preference during domestication (Yu et al. 2008). Unlike ssp. *japonica* rice, nonfunctional *Phr1* alleles were not preferentially selected in ssp. *indica* rice, possibly because opposing selection pressures favoring PPO activity in the tropical and subtropical regions where ssp. *indica* varieties thrive, potentially linked to the selection for improved disease resistance and seed dormancy (Yu et al. 2008). Moreover, a study (Gross et al. 2009) found that 96% of weedy red rice in the United States displays a positive response to phenol solution, and other samples exhibiting a negative reaction carry rare loss-of-function alleles, particularly a 1-bp frameshift deletion in exon 3 of *Phr1* gene.

In African rice (*O. glaberrima*), the genetic basis of hull color remains poorly characterized compared to Asian rice (*O. sativa*), despite its independent domestication history. Although *Phr1* has been well studied in Asian rice, its variation and functional role in African rice remain unclear. It is also unknown whether *Phr1* underwent similar selective pressure as ssp. *japonica* varieties or whether it exhibits a different genetic mechanism relating with the phenol color response. Therefore, studying *Phr1* gene could provide the breeders with a novel allele for improving grain storage stability and reducing grain discoloration. It could also offer insights for understanding the evolutionary dynamics of domestication and for expanding the genetic resources available for rice improvement. In this study, the genetic variations in phenol color reaction *Phr1* gene in African rice were examined by comparing hull color variation and underlying genetic mechanisms between *O. glaberrima* and its wild ancestor *O. barthii*, and the role of human selection in shaping this trait during domestication was elucidated.

## Materials and Methods

### (1) Genetic Materials

All the seeds and DNA materials used in this study were obtained from the genebank of International Rice Research Institute except 21 lines of *O. barthii* which were obtained from National Institute of Genetics (NIG) Gene Bank, Japan. A total of 126 lines of *O. glaberrima* (Table S1) and 45 lines *O. barthii* (Table S2 and S3) were used for phenotyping. For genotyping, 21 lines (Table S3) were analyzed using next-generation sequencing (NGS), and 29 lines (Table S2) were analyzed using Sanger sequencing, including 5 lines that overlapped with those analyzed by NGS. The population F_2_ plants were generated from the cross between OG101 (a phenol positive line) and OG145 (a phenol negative line). The resulting F₁ plants were self-pollinated and F₂ individuals were grown under greenhouse conditions. The seeds obtained from F_2_ individuals were used for segregation analysis.

### (2) Phenol Color Reaction Analysis

To evaluate phenol induced color changes, three seeds for each plant were used for phenol color treatment. The grains that were still intact with their hulls were soaked in a 1.5% aqueous phenol solution (Gross et al. 2009) for four days, then dried on the tissue paper for approx. 2 min and compared to untreated grains from the same plants to evaluate the color changes. Black color change after the phenol treatment was regarded as a phenol positive phenotype and no color changes represented a phenol negative phenotype. All phenotyping assays included IR64 (*O. sativa* ssp. *indica*) as a positive control and Kitaake (*O. sativa* ssp. *japonica*) as a negative control. Samples exhibiting a negative phenol reaction were phenotyped at least twice to confirm the result.

### (3) Genotyping Analysis of the Phr1 Gene

Young leaf tissues of *O. glaberrima* (126 lines), *O. barthii* (29 lines) and F_2_ population (40 lines) were used for DNA extraction with TPS buffer. To genotype the 1-bp deletion (chr04: 31,749,541) in the exon 1 of *Phr1* gene, which was formerly found from sequence alignment observation, one primer pair was designed to cover the targeted region from 31,749,245 to 31,749,751 in the chromosome 4 of *Phr1* sequence: Phr1_Gla_Indel1_Fw (5ʹ-GTGCATCACCAACCTCCAGA-3ʹ) and Phr1_Gla_Indel1_Rv (3ʹ-GGGAAGAAGAGCCAGCAGCT-5ʹ). PCR was carried out in a 50 µL reaction mixture containing 25 µL of KOD One™ PCR Master Mix (TOYOBO, Japan), 21 µL of PCR-grade water, 1.5 µL of 10 pmol/µL forward primer, 1.5 µL of 10 pmol/µL reverse primer, and 1 µL of genomic DNA. Thermal cycling was performed under the following conditions: initial denaturation at 98 °C for 1 min, followed by 30 cycles of denaturation at 98 °C for 10 s, annealing at 60 °C for 5 s, and extension at 68 °C for 1 s; with a final hold at 10 °C. The amplified PCR products were then sequenced by the Sanger’s method.

### (4) Next Generation Sequencing Data Analysis

Publicly available raw sequencing data for *O. glaberrima* (126 lines) and *O. barthii* (21 lines) were downloaded in FASTQ format from the DNA Data Bank of Japan (DDBJ) Sequence Read Archive (DRA023531) and (DRR057970–DRR057990) respectively. Then, the raw sequencing reads of each line were processed to remove the low-quality base sequences and trimmed using fastp v0.23.4 (Chen et al. 2018). The preprocessing step was conducted within a Singularity container, and sequence alignment was performed on the National Institute of Genetics (NIG) supercomputer, employing *O. sativa* ‘Nipponbare’ IRGSP v1.0 reference sequence (Kawahara et al. 2013), which had been indexed using the BWA v0.7.8 (Li and Durbin. 2009). Sequence reads for each sample were aligned to the indexed reference genome using ‘bwa mem’ (Li. 2013). For the post-alignment processing, the resulting SAM files were converted to BAM format using Samtools v1.9 (Li et al. 2009), which was later sorted and indexed in IGV_2.16.2 software for data visualization (Robinson et al. 2011). PCR duplicates were identified and removed using Picard MarkDuplicates v2.26.4. (Broad Institute. 2019), with duplicate removal enabled and validation stringency set to LENIENT. Resulting BAM files were indexed using Samtools for downstream processing. Variant calling was performed using the HaplotypeCaller module from the Genome Analysis Toolkit (GATK) v4.2.2.0 (McKenna et al. 2010; Depristo et al. 2011), operated in gVCF mode to record both variant and non-variant sites. To facilitate joint genotyping, individual gVCF files were merged using GATK’s CombineGVCFs tool to generate the merged gVCF file that includes all the samples. To ensure the quality and reliability of the variant calls, the VCF file was filtered by excluding the variants with a quality score below 30, depth of coverage below 10, mapping quality score below 40, removing indels, and taking exactly two alleles per site. The *Phr1* region in *O. barthii* (chr04: 31,749,155–31,751,459) within the whole-genome variant call format (VCF) file was extracted by the ‘view’ function, implemented in bcftools v1.9 (Li. 2011) in the supercomputer. The desired *Phr1* region was also extracted from the *O. glaberrima* VCF file, which had been generated using the same preprocessing and variant calling pipeline. The three-dimensional structure of the *Phr1* proteins for MH63 (*O. sativa* ssp. *indica*) (Yu et al. 2008), Nipponbare (*O. sativa* ssp. *japonica*), OG101 (*O. glaberrima*) and OG 145 (*O. glaberrima*) were predicted by homology modeling using the SWISS-MODEL server (Waterhouse et al. 2018) and the resulting PDB files were imported into PyMOL v3.1.6.1 (The PyMOL Molecular Graphics System) (Schrödinger and DeLano. 2020) for visualization.

### (5) Haplotype Network and Geographical Distribution

To determine the genetic relationship and geographical distribution, the *Phr1* region of *O. barthii* and *O. glaberrima* VCF files were used for the haplotype analysis. SNP positions are manually inspected in both VCF files and corresponding FASTA files are generated. The nucleotide sequences were aligned utilizing MUSCLE alignment (Edgar. 2004) within the MEGA software v11 (Tamura et al. 2021). Subsequently, the aligned data were employed to analyze haplotype groups in DNAsp v6.12.03 (Rozas et al. 2017). TCS network analysis (Clement et al. 2002) was conducted for *O*. *glaberrima* and *O. barthii* separately using PopArt software v1.7 (Leigh and Bryant. 2015) to infer the genetic relationships among the haplotypes. For geographical distribution, all of the *O. glaberrima* (126 lines) and *O. barthii* (45 lines) were categorized based on phenol reaction phenotype (positive or negative) and visualized using the 3D mapping function in Microsoft Excel.

### (6) Genetic Diversity Analysis

To investigate nucleotide diversity (π) between *O. glaberrima* and *O. barthii*, an 800 kb genomic region, encompassing 400 kb upstream and 400 kb downstream of the *Phr1* gene, was extracted from each respective VCF file. SNPs were filtered to include only biallelic sites with a minor allele frequency (MAF) ≥ 0.05 and the filtered region was subdivided into 10 kb windows to enable localized diversity analysis. Allele frequencies were calculated for each 10-kb window using the –freq function in VCFtools (Danecek et al. 2011). Then, nucleotide diversity (π) was estimated for each 10-kb window by summing 2pq across all biallelic SNPs and normalizing by the window length (10 kb). To assess whether the observed patterns were dependent on the choice of reference genome, an additional dot plot comparison was performed *O. sativa* ‘Nipponbare’ IRGSP v1.0 reference sequence and the corresponding *O. glaberrima* region (*Oryza_glaberrima*_V1; INSDC assembly GCA_000147395.1 was obtained from Ensembl Plants) used as the query genome. Pairwise sequence alignments were performed using NUCmer v3.1 from the MUMmer package (Kurtz et al. 2004). Dot plots were generated to assess collinearity between the 800 kb chromosome regions. In addition, segregation site, theta per site, nucleotide diversity per site, and Tajima’s D statistic for the combined dataset were calculated using the *Phr1* locus FASTA files of *O. glaberrima* and *O. barthii* in DNAsp v6 (Rozas et al. 2017). An additional 43 *Phr1* sequences previously published by Yu et al. (2008) were retrieved from GenBank and incorporated into the analysis. These included 8 *indica*, 14 *japonica*, and 30 *O. rufipogon* accessions.

## Results

(1) Variation of color response traits by phenol treatment across the samples

**Fig. 1:**
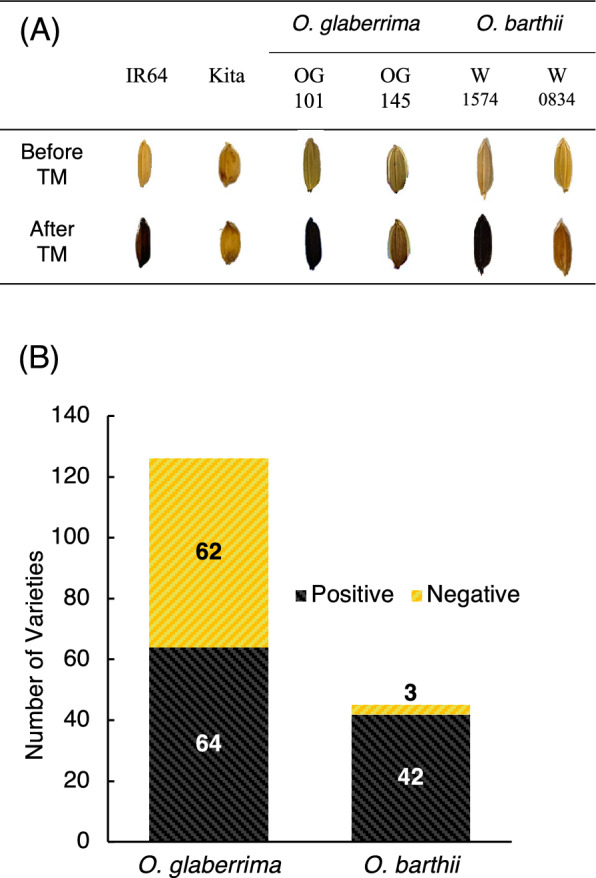
Phenol Chemical Assay in African rice. **A** Color changes after phenol response. Phenol positive control IR64 (*indica*), Phenol negative control Kitaake (*japonica*), *O. glaberrima* (OG101 and OG145) and *O. barthii* (W1574 and W0834). **B** Summary of the distribution of phenol positive and negative lines in *O. glaberrima* and *O. barthii* with the black-colored section indicating the proportion of phenol-positive individuals and the yellow-colored section indicating negative individuals

The positive control IR64 (*O. sativa* ssp. *indica*) and negative control Kitaake (*O. sativa* ssp. *japonica*) showed black color changes representing phenol positive phenotype and no color change referring to phenol negative response, respectively after the treatment (Fig. 1A). In *O. glaberrima*, 62 out of 126 lines exhibited no color changes, representing a negative phenol color reaction (Fig. 1B; Table S1). The seeds of the remaining 64 lines turned black as a phenol response, referring to a positive phenol color reaction. In *O. barthii*, 3 lines out of 45 lines showed a negative reaction except for the remaining samples, which all showed black color change (Fig. 1B; Table S2 and S3).

(2) Genotyping uncovers a newly discovered mutation associated with phenol non-responsive phenotype.

**Fig. 2:**
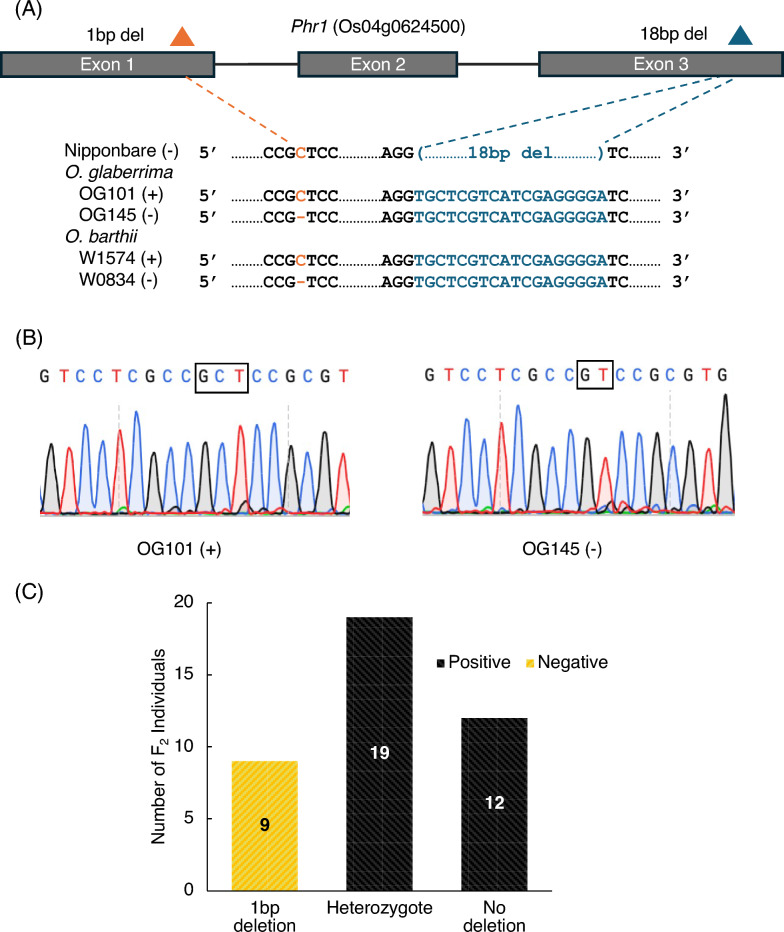
Variation of phenol color reaction gene (*Phr1*) between positive and negative lines in African rice. **A** Genotype comparison of functional and non-functional *Phr1* gene in *O. glaberrima* and *O. barthii* with reference sequence Nipponbare. ( +) represents phenol positive phenotype and (-) represents phenol negative phenotype. Orange and Aegean blue triangles represent 1 bp deletion and 18 bp deletion respectively. **B** Sanger sequencing results showing functional *Phr1* (OG101) with no deletion and non-functional (OG145) with 1-bp deletion. **C** Segregation analysis of phenol color response in the F₂ generation of a cross between functional *Phr1* (positive phenotype) line, OG101 and non-functional *Phr1* (negative phenotype) line, OG145. Observed allele frequencies were consistent with the expected 1:2:1 segregation ratio. Black and yellow bars indicate phenol positive and negative phenotypes respectively

Yu et al. (2008) reported that the phenol color reaction of *O. sativa* is controlled by the gene *Phr1*. The majority of ssp. *japonica* varieties of *O. sativa* contain an 18-bp deletion in exon 3, while a minority exhibit either a 29-bp deletion in exon 3 or a 1-bp insertion in exon 1 (Yu et al. 2008), and another rare mutation found in weed accession red rice involves a 1-bp deletion in exon 3 in the *Phr1* gene (Gross et al. 2009). To understand the difference of the phenol reaction observed in African rice varieties (Fig. 1), the sequences of the *Phr1* were surveyed using two phenol positive varieties (OG101, *O. glaberrima* and W1574, *O. barthii*) and two phenol negative varieties (OG145, *O. glaberrima* and W0834, *O. barthii*) (Fig. 2). Comparing to the Nipponbare sequence (IRGSP1.0) which carries an 18-bp deletion in exon 3, all four African rice varieties had 3-bp deletion and 18-bp insertion in the exon 1 and 3, respectively, (Figure S1). The apparent 18-bp insertion is relative to the 18-bp deletion present in the Nipponbare (ssp. *japonica*). Because the other 3-bp deletion was observed in both phenol positive and negative varieties, it was concluded that it does not affect the phenol reaction difference (Figure S4). In addition to these two InDels, it was found that two phenol negative varieties had 1-bp deletion in exon 1 (chr04: 31,749,541), which has not been found in previous studies (Fig. 2A and B). To confirm genetic association between this 1-bp deletion and phenol reaction phenotype, both NGS and Sangar sequence data on the *Phr1* locus of African rice varieties were analyzed. It was found that 1-bp deletion mutation is observed only in phenol negative varieties but not in phenol positive ones (Table 1). These results suggested that 1-bp deletion in the exon 1 of the *Phr1* locus was the causative mutation site which can cause the phenol reaction difference. This deletion causes a frameshift mutation, which alters the amino acid sequence and introduces a premature stop codon (Figure S2), resulting in the loss of functional *Phr1* protein and a phenol-negative reaction. The predicted 3D protein structure of functional and non-functional *Phr1* (Figure S3) further supports the disruption of the functional conformation.

**Table 1:** Phenotypic expression of phenol color reaction corresponding to genotypic variation

	1 bp deletion	Deletion not detected
*O. glaberrima*		
Positive	0	64
Negative	62	0
*O. barthii*		
Positive	0	42
Negative	3	0

(3) Segregation of F_2_ population shows Mendelian ratios.

For the further confirmation of the effect of the 1-bp deletion on the phenol reaction phenotype, the F_2_ population (40 plants) was developed from the cross between phenol positive (OG101) and negative (OG145) varieties. The phenol reactions of the grains produced by the F_2_ plants were then investigated. Among the 40 F_2_ plants, 31 showed phenol positive phenotype while the other 9 showed negative. Segregation ratios were tested for goodness-of-fit to expected Mendelian ratios using a Chi-square (*X*^*2*^) test (Snedecor and Cochran 1989) and the result indicated that segregation of the phenol-responsive phenotype in the F_2_ population did not deviate significantly from the expected 3:1 Mendelian ratio (*X*^*2*^ = 0.133, *df* = 1, *p* = 0.715), consistent with single-gene inheritance of the phenol reaction (Fig. 2C). Interestingly, all the grains that showed black color changes after the phenol treatment initially displayed a mixed yellow and black coloration since before the treatment (during seed maturation). It was then surveyed the genotypes of the F_2_ plants. All 31 F_2_ plants whose genotypes are homozygous for the non-deletion allele and heterozygous showed phenol positive reaction. The remaining 9 plants whose genotype is homozygous for the 1-bp deletion exhibited no color change. Segregation of the genotypes of the *Phr1* locus (9:19:12) approximated the expected 1:2:1 Mendelian ratio. These results showed a strong association between genotype and phenotype.

(4) Genetic relationship at *Phr1* locus revealed by haplotype analysis in African rice.

**Fig. 3:**
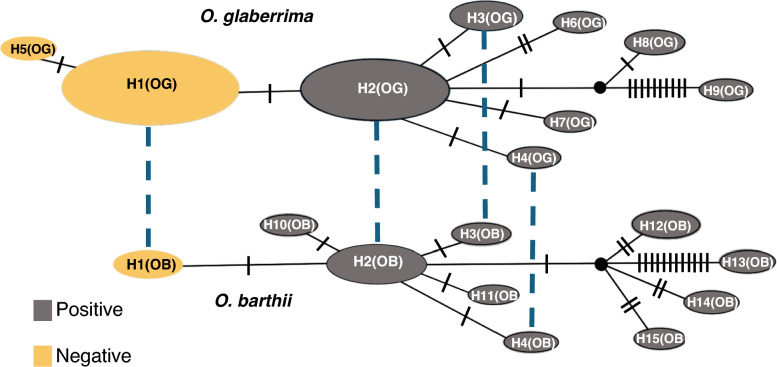
Haplotype network analysis for African rice. TCS network algorithm based on the region of *Phr1* locus. A dotted blue line connects identical haplotypes between the two populations, *O. glaberrima* and *O. barthii*. Each haplotype is represented by a circle, the size of which is proportional to the number of samples. Black lines and numbers on the branches are used to indicate the number of mutational changes between two different haplotypes. Black and yellow colors indicate phenol positive and negative haplotypes respectively

To understand how the 1-bp deletion in the *Phr1* gene arose and maintained in African rice varieties, the haplotypes observed in *O. glaberrima* and *O. barthii* were compared. In *O. glaberrima*, a total of 9 distinct haplotypes (Fig. 3) has been identified in the *Phr1* gene across 126 varieties (detailed haplotype list is presented in Table S4). Among these haplotypes, phenol negative varieties with 1-bp deletion were associated with two unique haplotypes, haplotype 1 (H1OG) with largest variety number (n = 61) and haplotype 5 (H5OG) with only one variety (n = 1). These two haplotypes differ by only a single nucleotide. In *O. barthii*, 10 diverse haplotypes are present within 21 varieties including only one negative haplotype (haplotype 1, from this point forward referred as H1OB (n = 2)). Interestingly, sequences of the most frequent negative haplotype in *O. glaberrima*, H1OG is identical to that of the negative haplotype 1 (H1OB) found in *O. barthii*, suggesting a shared genetic origin or potential gene flow between *O. glaberrima* and *O. barthii*. In addition, three positive haplotypes in *O. glaberrima*, including the major positive haplotype 2 (H2OG) (n = 50) and two additional positive haplotypes, haplotype 3 (H3OG) (n = 3) and haplotype 4 (H4OG) (n = 1) were identical to three positive haplotypes, major positive haplotype 2 (H2OB) (n = 9), haplotype 3 (H3OB) (n = 1) and haplotype 4 (H4OB) (n = 1), respectively, in *O. barthii*. Each of these haplotypes within the same species differs by only a single nucleotide, suggesting recent divergence from a common ancestral haplotype. Here, a pivotal question arises: which of these haplotypes served as the cradle for the emergence of negative haplotype in African rice? With the idea that *O. barthii* is the ancestral species of *O. glaberrima*, it is suggested that major positive haplotype, haplotype 2 (H2OB) in *O. barthii,* played this crucial role, where the *Phr1* gene experienced a loss-of-function mutation (1-bp deletion), ultimately giving rise to the novel haplotype 1 (H1OB). However, another possibility exists; the phenol-negative haplotype with 1-bp deletion arose in *O. glaberrima* and transferred to *O. barthii* through gene flow between two species.

**Fig. 4:**
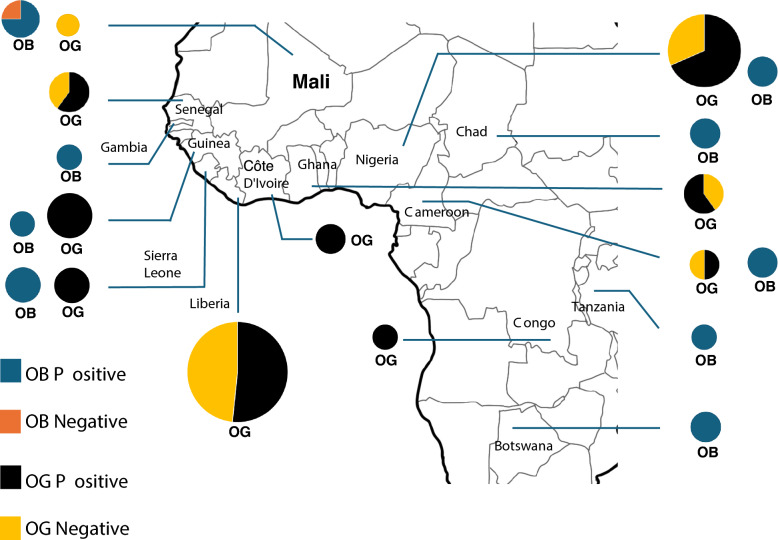
Geographic distribution of *Phr1* positive and negative groups in African rice population. The pie charts in the map indicate the frequency of the individual phenotype in each country and the size of pie corresponds to the number of individuals. For *O. barthii*, aegean blue and orange colors represent phenol positive and negative phenotypes respectively. For *O. glaberrima*, the black and yellow colors correspond to positive and negative respectively

Regarding the geographical distribution of *O. glaberrima*, both phenol positive and negative varieties were observed in Liberia, Nigeria, Ghana, Cameroon, Gambia, and Côte d'Ivoire, whereas only the negative variety was detected in accessions from Mali (Fig. 4). In *O. barthii,* only the negative variety was exhibited in Mali. Interestingly, both species share the identical negative haplotype (OBH2 and OGH2) in this country.

(5) Reduced nucleotide diversity indicates selective pressure on phenol non-responsive phenotype.

To examine whether alleles of the *Phr1* gene are selected during domestication and varietal diversification processes of *O. glaberrima*, the nucleotide diversity of *Phr1* gene was analyzed. In Asian rice gene pool, *O. rufipogon* exhibits the highest nucleotide diversity of 0.00614 compared to the other species (Table 2). In *O. sativa* (both in ssp. *indica* and *japonica*), nucleotide diversity was lower in ssp. *japonica* varieties with the negative phenotype (loss-of-function of the target gene) than in ssp. *indica* varieties with the positive phenotype (functional allele). Interestingly, levels of nucleotide diversity in *O. barthii* are much lower, having 0.00118 in *Phr1* locus, followed by *O. glaberrima*, the lowest with 0.00016. To check whether the selection process occurred in phenol positive varieties or negative varieties in the African rice, the nucleotide diversity in both positive and negative varieties was compared. In *O. barthii*, the sample size for the negative group is small with n = 2, and both of them have identical *Phr1* regions, so there are no parameters for it. In *O. glaberrima*, it was found that the negative group has lower π value (0.00001) compared to the positive varieties (0.00029), suggesting the evidence of selection process in negative ones.

**Table 2:** Genetic Diversity *Phr1* locus in different varieties of rice

	*Phr1* locus			
Taxon			Nucleotide	Tajima's D
	Segregation	Theta	Diversity	
	Site	(per site)	per site (Pi)	
*O. rufipogon* (n = 30)	57	0.00692	0.00614	-0.42602
*O. sativa* (n = 22)	37	0.00446	0.00387	-0.60967
*indica* ( +) (n = 8)	28	0.00469	0.00562	1.04265
*japonica* (-) (n = 14)	28	0.00401	0.00246	-1.66102
*O. barthii* (n = 21)	22	0.00263	0.00118	-2.08321
*O. barthii* ( +) (n = 19)	22	0.00271	0.00130	-2.01916
*O. barthii* (-) (n = 2)	0	0	0	0
*O. glaberrima* (n = 126)	18	0.00140	0.00016	-2.46814
*O. glaberrima* ( +) (n = 64)	17	0.00160	0.00029	-2.43659
*O. glaberrima* (-) (n = 62)	1	0.00009	0.00001	-1.08044

**Fig. 5:**
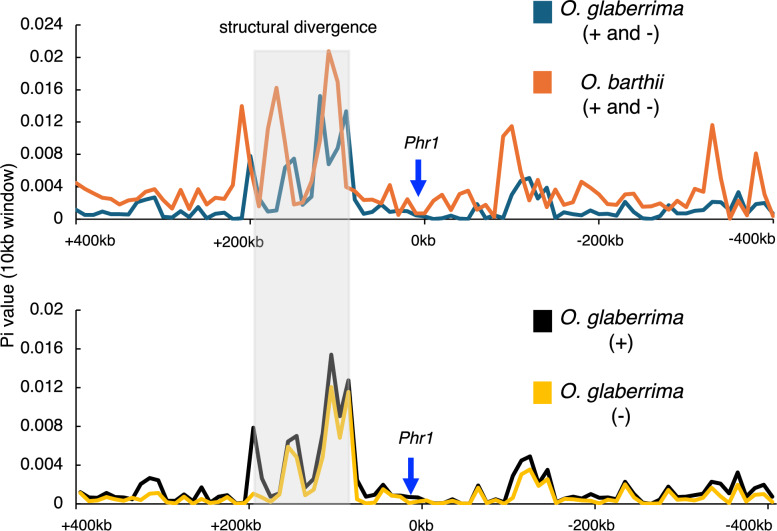
Comparison of nucleotide diversity pattern of *Phr1* gene. The nucleotide diversity was calculated using 10 kb sliding window across 400 kb downstream and 400 kb upstream regions of *Phr1* locus in *O. glaberrima* and *O. barthii* were analyzed. The blue arrow was used to indicate the 10 kb region which includes *Phr1* locus. Aegean blue and orange lines represent *O. glaberrima* and *O. barthii* lines, respectively. Black and blue lines represent positive and negative lines of *O. glaberrima*, respectively. The grey box area represents the regions with unstable nucleotide diversity and poor collinearity with the reference genome

Additionally, to investigate the nucleotide diversities in the surrounding chromosomal region, π values in the 400 kb upstream and downstream of the *Phr1* gene (total 800 kb) were analyzed using a window size of 10 kb with no overlap (step size = 10 kb). Nucleotide diversity (π) was generally higher in wild rice (*O. barthii*) compared to cultivated rice (*O. glaberrima*) across the analyzed chromosomal region. This pattern is consistent with the expected reduction in genetic diversity resulting from domestication process. Within the 800 kb interval, π fluctuations were confined to specific regions which were highlighted by a grey box in Fig. 5. The dot plot analysis using two species’ genome sequences revealed strong collinearity between *O. sativa* (Nipponbare) and *O. glaberrima*, indicating conserved gene order across most of the 800 kb region (Figure S5). Several small reverse-orientation alignments were also observed, suggesting the presence of minor local inversions or rearrangements, but no large-scale structural variation between *O. sativa* and *O. glaberrima* was detected. In comparison of the positive and negative varieties in *O. glaberrima*, no clear differences of nucleotide diversity were observed in the surrounding chromosomal regions (Fig. 5), showing no evidence of the genetic hitchhiking effect by the *Phr1* gene.

## Discussion

The negative phenol color reaction in Asian rice varieties is shown to be associated with *Phr1* mutations due to indels in the coding regions, three mutation sites in ssp. *japonica* cultivars (Yu et al. 2008) and one in red rice (weedy rice) accession (Gross et al. 2009). In this study, 1-bp deletion in the exon 1was identified that causes frame shift of amino acid and loss-of-function in the *Phr1* in both wild and domesticated African rice. In addition to the 1-bp deletion, 3-bp deletion was found in exon 1 of *Phr1* gene in almost all African rice. In the case of 3-bp deletion, it does not affect the phenotype of phenol reaction (Figure S1), suggesting that this 3-bp deletion does not have a significant impact of the function of *Phr1*. While it was proposed that African rice originated from Asian rice via sympatric speciation by Nayar (1973, 2010, 2012, 2014), genetic evidence (Huang et al. 2015) and domestication timing (Sweeney and McCouch. 2007) support an independent origin of African rice from Asian rice. In the present study, it was further observed that independent mutations of *Phr1* gene in African (*Oryza glaberrima*) and Asian (*Oryza sativa*) rice, arising separately after their divergence rather than inheritance of a common allele. To confirm the association between 1-bp deletion and phenol negative phenotype, segregating F_2_ population was developed deriving from the cross between phenol positive and negative varieties. The result showed that all homozygotes for the non-deletion allele and heterozygotes F_2_ plants showed phenol positive reaction, indicating that this 1-bp deletion is the causal factor for the phenol color reaction in African rice species.

The geographical distribution of the haplotypes of African rice provides a clue about where this 1-bp deletion rose in Africa. Analysis of whole genome sequences of *O. glaberrima* and *O. barthii* suggested that the exact domestication center was inland Niger Delta and specifically Northern Mali (Cubry et al. 2018). In this study, the analysis showed that the major positive haplotype (H2OB) of *O. barthii* was distributed in many countries, including Sierra Leone, Guinea, Mali, Gambia, Cameroon, Chad and Nigeria. Similarly, the major positive haplotype of *O. glaberrima* (H2OG) is identical to that of *O. barthii* (H2OB) and distributed in many regions. However, the negative haplotype (H1OB) in *O. barthii* that contains 1-bp deletion was found only in Mali and interestingly, the identical negative haplotype (H1OG) of *O. glaberrima* was also found in Mali. This striking similarity suggests a potential scenario; a major positive haplotype (H2OB) in *O. barthii* is the primary haplotype in African rice species, from which the loss of function mutation of 1-bp deletion in the *Phr1* gene arose in Mali. This mutation was later inherited by *O. glaberrima* in Mali and was distributed to other regions, including Liberia and Nigeria, sharing the same haplotype. This finding contributes to our understanding of genetic variation in *O. glaberrima* and shed light on the evolutionary dynamics of the *Phr1* gene. Another negative haplotype (H5OG) in *O. glaberrima* is likely derived from the major negative haplotype (H1OG) of *Phr1* gene.

To evaluate whether this mutation has been subject to selection, we examined nucleotide diversity across different rice populations at this *Phr1* locus. The nucleotide diversity of *O. glaberrima* in *Phr1* locus is the lowest compared to different rice species and this reduced genetic diversity may be due to a domestication bottleneck. Significantly low nucleotide diversity value of *Phr1* locus was observed in the negative group in *O. glaberrima* although the positive group maintains higher genetic diversity with six distinct haplotypes. These results suggested that the alleles for the negative phenotype were selected against the positive ones in the domesticated African rice. The significantly negative Tajima’s D value of *Phr1* locus in the negative group also supports this scenario. In contrast, in terms of the wide range of 800 kb genomic regions, the pattern of nucleotide diversity value in negative varieties is not consistently low, with elevated values observed in certain regions flanking the target gene. This pattern may indicate historical recombination events during the evolutionary process.

The results of genetic diversity suggested that accessions carrying the negative allele are attributable to selection in cultivated African rice (*O. glaberrima*). This rises a new question: to what extent is grain discoloration in rice influenced by *Phr1* allele? The parental phenol-positive line OG101 and phenol-negative line OG145 both exhibited yellow hulls, whereas most of the seeds of the homozygous and heterozygous F₂ plants displayed a half black–yellow hulls (Figure S6). Although rice hull color change during seed development is typically associated with the *Bh4* (*Black hull 4*) gene (Zhu et al. 2011), our analysis of phenotypic and genotypic segregation ratios does not support a complementary gene model (*Bh4* and *Phr1*) and the strong association between phenotype and *Phr1* genotype indicates that *Bh4* is unlikely to be involved in this trait. The appearance of grain discoloration in phenol positive lines of F_2_ plants may be influenced by environmental factors which induce the polyphenol oxidation during the seed development stage. This is further supported by our observations in the greenhouse where grain discoloration, although occurring only in certain cultivation years, is easily noticeable. However, the specific environmental factors contributing to this trait remain unknown. This intermittent but visible color change would allow farmers to identify and select against the negative group in the field, even without chemical testing.

In wheat, the reduction of grain discoloration has been a key breeding objective due to consumer's preference for white grains (Anderson and Morris. 2001). This may also be applied to rice, where the economic importance of grain appearance could lead farmers to prefer lines, such as the negative group, that exhibit stable hull coloration and reduced discoloration during maturation. Most importantly, if the *Phr1* gene in ssp. *japonica* rice shows sign of positive selection (Yu et al. 2008), it is plausible that a similar selection pressure acted on the negative haplotype in African rice varieties, favoring traits such as stable hull coloration during domestication. This hypothesis was supported by the haplotype analysis in *O. glaberrima*, where the half of the samples showed same negative haplotype.

In *O. barthii*, no clear selection patterns is detected since the sample size for phenol negative lines is very small, bearing only three individuals. Hereby two hypotheses were developed. One possible explanation is that the novel mutation arose in wild ancestor *O. barthii* and later inherited by the *O. glaberrima* during domestication. Another possibility is the gene flow between weedy rice and cultivated rice where a novel mutation arose within the negative group of *O. glaberrima* and was subsequently favored by farmers. This mutation may have introgressed into *O. barthii*, which could account for the relatively small number of negative accessions observed in the wild population.

The functional *Phr1* gene in ssp. *indica* rice is believed to represent the counterbalance selection of polyphenol oxidase (PPO) disease resistance and enhanced seed dormancy in tropical and subtropical climates (Yu et al. 2008). In this study, most of the *O. barthii* samples were originated from tropical and subtropical regions of Africa. In *O. barthii*, 48 out of 50 lines exhibit the functional allele, indicating a substantial prevalence of this allele under the prevailing environmental conditions. On the other hand, in *O. glaberrima*, there is a near-equal ratio of functional to non-functional gene variants, suggesting a well-balanced distribution of gene variants in response to the specific environmental conditions in these regions. In addition, it is suggested that long awns and black hulls in rice enhance survival by reducing detection and predation by birds in the dark mud substrates of rice fields (Anderson and Morris. 2001). This also supports that the positive group is more favorable in wild African rice.

Overall, this study contributes to our understanding of the evolutionary history of the targeted *Phr1* gene in African rice and independent domestication of *Phr1* gene in Asian and African rice, highlighting how domestication and selective breeding may have influenced patterns of genetic diversity. In addition, this study can ultimately aid in more targeted and efficient rice plant breeding efforts, leading to the development of improved varieties with desired traits such as stable hull color, enhanced disease resistance and increased productivity.

## Conclusion

The phenol color reaction, regulated by the *Phr1* gene, serves as a significant biochemical marker for evaluating genetic variation in rice. A new mutation, 1-bp deletion in exon 1 of *Phr1* gene, which is associated with the phenol reaction phenotype in African rice, was identified. Our findings indicated that the non-functional allele in *O. glaberrima* which corresponds to phenol negative lines was favored during domestication and breeding, but functional alleles remain in the phenol positive lines, preserving genetic variability. Our results also showed that phenol negative phenotype was independently selected from Asian rice during domestication. In addition, the geographic and genetic distribution of *Phr1* gene provides insights into the selection pressures exerted by past cultivation practices and reveals aspects of the evolutionary history of rice in Africa.

## Availibility of Data and Materials

We use publicly available data set for *O. barthii* (21 lines) from DDBJ website (Accession number DRR057970–DRR057990), for *O. glaberrima* (126 lines) (Accession number DRA023531) and *O. rufipogon* and *O. sativa* from GenBank under accession numbers DQ532375–DQ532429.

## Supplementary Information

Additional file1 (DOCX 1859 KB)

Additional file2 (XLSX 19 KB)

## Data Availability

We use publicly available data set for *O. barthii* (21 lines) from DDBJ website (Accession number DRR057970–DRR057990), for *O.glaberrima* (126 lines) (Accession number DRA023531) and *O. rufipogon* and *O. sativa* from GenBank under accession numbers DQ532375–DQ532429.
