# Supplementary material for: Independent Origin of Phenol Non-responsive Phenotype Caused By Phr1 Variation During Domestication of Asian and African Rice
Source: Rice (N Y). 2026 Jan 22;19:13. doi: 10.1186/s12284-026-00884-x (PMC12909727; doi:10.1186/s12284-026-00884-x)
Supplement: Supplementary file 1 — Additional file1 (DOCX 1859 KB) [file 12284_2026_884_MOESM1_ESM.docx]

Figure S1. Schematic diagrams of *Phr1* gene in diffference rice species illustrating the functional (Blue box) and non-functional (Grey box) gene. (+) denotes a phenol-positive response and a functional *Phr1* gene, while (-) indicates a phenol-negative phenotype associated with loss-of-function mutations. The introns are depicted as connecting lines within the diagrams.

Figure S2. Alignment of *Phr1* protein sequences of non-functional OG145 (*O. glaberrima*) with functional MH63 spp. *indica* and non-funcitonal Nipponbare (Nip) ssp. *japonica*. Putative N-terminal signal peptide and tyrosinase copper binding domains (1 and 2) are indicated in pink and orange, respectively. The frameshift amino acid change is highlighted in red, an asterisk (*) denotes a stop codon and dots following the stop codon denote absence of amino acids. Deletion sites of 3-bp in OG145 and 18-bp in Nipponbare is highlighted in blue and yellow, respectively.

Figure S3. Predicted protein 3D structural differences between functional and non-functional *Phr1* in Asian and African rice.

Figure S4. Structural superposition of funcitonal *Phr1* protein models between the *O.glaberrima* (OG 101) and *O. sativa* (MH63) were visualized in PyMOL and highlighted in palecyan and light pink respectively. *O.glaberrima* (OG 101) protein lacks a proline residue due to a 3-bp deletion in the coding sequence, while *O. sativa* (MH63) retains this residue. This structural differences are highlighted in blue and pink.

Figure S5 . Dot plot comparison of a 800kb of chromosome 4 region between *O. sativa* ssp. japonica (Nipponbare) and *O. glaberrima*. Genomic region of 800kb alignments were generated using NUCmer and filtered with delta-filter -r -q to retain the best reciprocal matches. Each dot represents an aligned sequence block. Blue dots indicate alignments in the same orientation, while red dots indicate reverse-orientation alignments. The strong diagonal pattern indicates overall collinearity between the two genomes, with minor local rearrangements. The grey box highlights a region exhibiting localized structural variation.

Figure S6. Grain color phenotypes observed in the F₂ population grown under greenhouse conditions. Homozygous deletion plants show yellow grains, whereas heterozygous and homozygous wild-type plants exhibit partially black grains.
